# Concrete mix design and aggregate tests data between 2009 and 2017 in Sudan

**DOI:** 10.1016/j.dib.2018.09.061

**Published:** 2018-09-26

**Authors:** Amged O. Abdelatif, Amir M.Y. Shaddad, Mohammed B. Fathallah, Mohammed S. Ibrahim, Mohammed H. Twfeeq

**Affiliations:** Department of Civil Engineering, Faculty of Engineering, University of Khartoum, Sudan

## Abstract

Over 1000 mix designs data and more than 1400 fine and coarse aggregates tests results data were collected for the previous 10 years in Sudan. The data was organized into spreadsheets creating a data base useful for future studies. The data includes mix designs proportions in terms of cement content, water, fine and coarse aggregate in addition to the used admixtures. Moreover, the data provide the gained concrete strength in 7 and 28 days for those mix designs. The data also includes properties of aggregates such as aggregate type, maximum size, percentage passing sieve 0.6 mm, and silt/clay/dust contents. The data are hosted in Mendely data: Abdelatif et al. (2018).

**Specifications table**

**Table:** 

Subject area	*Civil Engineering, Structural Engineering, Building Materials,*
More specific subject area	*Building Materials, Concrete Technology*
Type of data	*Tables*
How data was acquired	*Data mining of previous concrete mix design trials and aggregate tests*
Data format	*Raw, Analyzed*
Experimental factors	*Brief description of any pretreatment of samples*
Experimental features	*Very brief experimental description*
Data source location	*All of the data are acquired from the University of Khartoum Material laboratory in the Civil Engineering Department, the leading laboratory in Sudan for material testing.*
Data accessibility	*Mendeley data: Abdelatif, Amged; Shaddad, Amir; babiker, mohammed; Saeed, Mohamed; Hisham, Mohamed (2018), “Grade 25 and 30 of Concrete mix design and aggregate tests data between 2009 and 2017 in Sudan”, Mendeley Data, v1*
	http://dx.doi.org/10.17632/gp2nvxn6zg.1

**Value of the data**

• No current dataset for previous concrete mix designs or aggregate tests in Sudan are provided.
• The data could be used either by researchers in future researches or by the engineers in the construction industry in Sudan.
• This data also may encourage other researchers to share their dataset to enrich the information needed to the research in Sudan.
• Comprehensive statistical analysis could be carried out to yield with more valuable information.
• This data gives an insight into the concrete mix designs and aggregates tests in Sudan and their credibility.
• The data can help improve concrete casting in Sudan.

## 1 Data

The data was from University of Khartoum Concrete and Materials Laboratory. More than 1000 mix designs of grade 25 and 30 concrete for the previous ten years were collected. In addition over 1400 fine and coarse aggregates tests for the same period of time were included as well. All the data was organized and categorized. All the raw data are available in [1], and it can be used for possible future researches as a data base or benchmark.

## 2 Experimental design, materials and methods

**Fig. 1:**
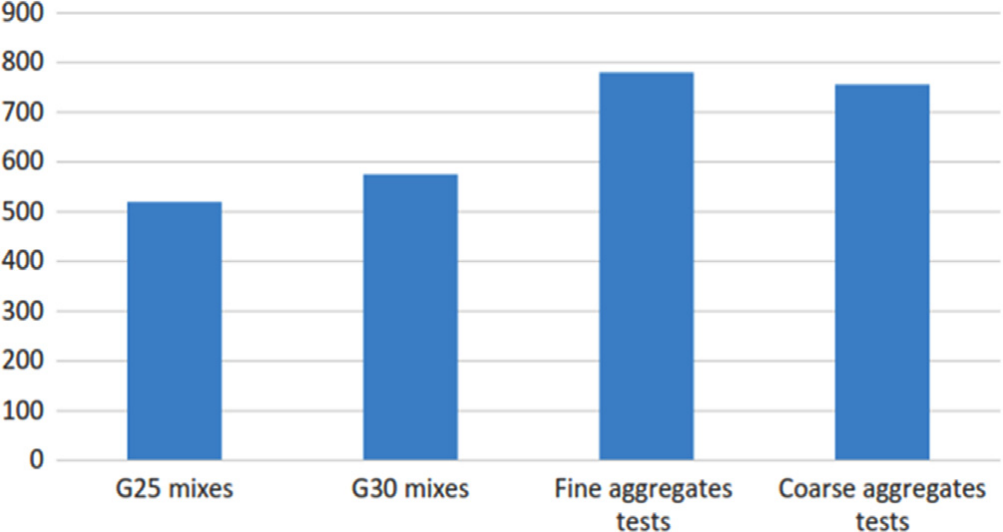
Number of mix design and aggregate tests.

The mix design data was categorized into grade 25 and grade 30 concrete. Fine and coarse aggregates was also entered into spreadsheets. The grade 25 and 30 mixes were over 500 mix for each grade. More than 700 test result for the fine aggregates and coarse aggregates each. The data are summarized in Fig. 1.

The following procedure was followed for the data entry for both concrete mix design and aggregate tests:

### 2.1 Concrete mixes spreadsheet

The concrete mixes for grade 25 and 30 are organized in spreadsheets including the following data:

• Date of mix
• Location were the mix was casted if available
• Type of fine and coarse aggregates used
• Maximum size of coarse aggregates
• Percentage of aggregates passing sieve 0.6 mm
• Target mean strength
• Cement content (O.P.C)
• Water cement ratio (w/c)
• Water content
• Additive type and dosage if used
• Total amount of aggregates
• Amount of fine and coarse aggregates separately
• Workability slump
• Hardened concrete density
• Strength in 7 days
• Strength in 28 days

### 2.2 Aggregates tests spreadsheet

**Fig. 2:**
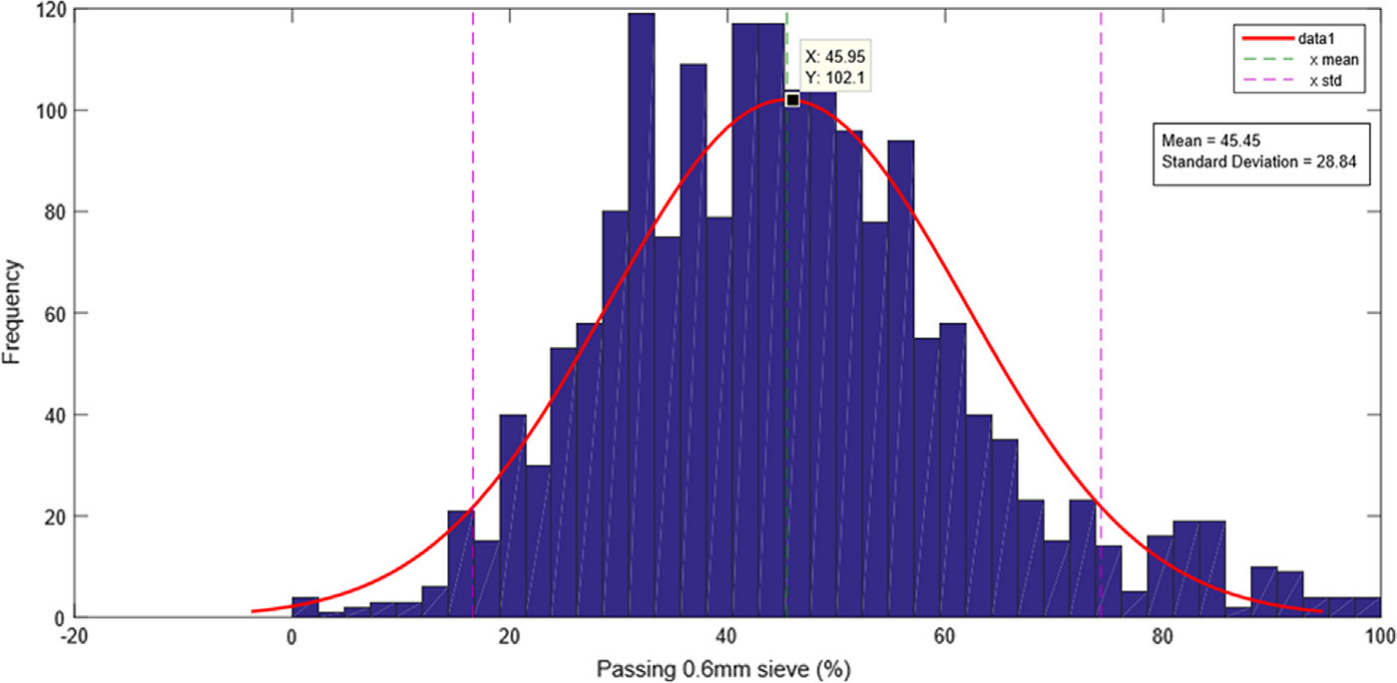
Percentage of fine aggregates passing 0.6 mm sieve histogram and normal distribution curve.

The fine aggregates data spreadsheets mainly contained the date and location of the aggregates if available. It also contained the type of aggregates, percentage passing sieve 0.6 mm, and the dust, silt and clay content. The percentage of aggregates passing sieve 0.6 mm using the mean of the mixes and fine aggregates data is 45.45% as shown in Fig. 2.

The coarse aggregates data entry is classified according to the type of aggregate in addition to the date and location. Most importantly it consists of the aggregates crushing value and impact value.
