# Supplementary material for: Concrete mix design and aggregate tests data between 2009 and 2017 in Sudan
Source: Data Brief. 2018 Sep 26;21:146–9. doi: 10.1016/j.dib.2018.09.061 (PMC6186963; doi:10.1016/j.dib.2018.09.061)
Supplement: Supplementary file 1 — Supplementary material [file mmc1.docx]

**Conflict of interest**

All the authors confirm as No conflict of Interest.
